# The influence factors of tour guides' professional identity and professional decision before and after the COVID-19 pandemic

**DOI:** 10.1016/j.heliyon.2024.e31588

**Published:** 2024-05-21

**Authors:** Wenwen Hu, Qing Yuan, Yaxi Wang, Nan Chen

**Affiliations:** a School of Management, Guangzhou College of Technology and Business, Guangzhou, 510850, China; b School of Cultural Industry & Tourism Management, Henan University, Kaifeng, 475001, China; c Research Institute for Study Travel, Henan University, Kaifeng, 475001, China

**Keywords:** Tour guides (TGs), Professional identity (PI), Tour guide professional identity (TGPI), COVID-19 pandemic, Profession decisions, Influence factors, Binary logistic regression

## Abstract

The COVID-19 pandemic has significantly impacted the tourism sector, particularly tour guides (TGs), affecting their professional identity (TGPI) and intentions to return to work. As China strives to revive its tourism industry, it is crucial to understand the current state of TGPI, its evolution, influencing factors, and its impact on TGs' return intentions. This study employed a quantitative approach, using comparative analysis and binary logistic regression, to investigate these issues among frontline TGs in China, pre- and post-pandemic. Cross-sectional surveys were conducted with 422 participants in 2019 and 398 in 2022, yielding 370 and 342 valid responses, respectively. The questionnaire utilized a five-point Likert scale. Findings reveal that (1) The overall TGPI level in 2022 post-pandemic is medium (3.93), showing a significant decrease from the pre-pandemic level in 2019 (4.15). (2) Influencing factors of TGPI are predominantly material, reflected in social insurance and income changes pre- and post-pandemic. (3) This study presents a novel definition and scale of TGPI, encompassing tour guides' professional value identity (TGPVI), emotion identity (TGPEI), relationship identity (TGPRI), and behavior tendency (TGPBT). (4) The two dimensions of the TGPI, TGPVI and TGPRI, income and education level, significantly influence TGs' return intentions. The study provides valuable academic and practical insights into TGPI and offers significant implications for enhancing TGs' return intentions and policymaking for post-pandemic tourism industry development.

## Highlights

• Explored the changes and the influence factors on the tour guides' professional identity (TGPI) before and after the COVID-19 pandemic.
• Developed a definition and scale of TGPI, according to their practical work.
• Explored the influence factors of tour guides' professional decision to return to their post after the pandemic.
• Offers practical and managerial implications for both tourism businesses and government departments.

## 1 Introduction

The COVID-19 pandemic has significantly impacted various tourism sectors [1,2], making it one of the hardest-hit industries [3,4]. The pandemic has also profoundly affected staff in the tourism and hotel industry [5], particularly tour guides (TGs) in China [6]. As many countries have begun to ease pandemic-related restrictions and gradually restore their tourism sectors, the Chinese government is also adjusting its policies to resume tourism. TGs, being crucial in enhancing tourist satisfaction and experience [7,8], play a vital role in tourism development. However, the current state of tour guide professional identity (TGPI) and their intention to return to the sector after prolonged separation due to the pandemic require urgent and serious consideration for the smooth recovery and development of China's tourism industry post-pandemic.

Professional identity (PI) is a critical variable in assessing individuals' professional states and plays a pivotal role in understanding individuals' professions and professional decisions [9]. PI is significant for both the individual's development and the profession itself. For individuals, PI significantly impacts learning engagement [5] and work engagement [10], directly affecting a person's career aspirations. Furthermore, PI strongly correlates with burnout and emotional well-being in the workplace [11,12], positively affecting job satisfaction [13]. For the profession, PI positively impacts job retention [14,15] and negatively affects turnover intention [16].

PI has garnered considerable attention due to its crucial role, leading to extensive discussions across various disciplines, including social science, medicine, management, communication, and information sciences. In the tourism sector, the role of PI is more prominent as it is a labor-intensive service industry, efficiently triggering burnout and professional mobility among practitioners [5]. Wang et al. [13] [][13][]argued that PI positively impacts employee engagement and satisfaction and negatively impacts turnover intention. Some scholars have argued that PI is critical in undertaking talent recruitment and retention in the hospitality industry [13,17], relating closely to the success and sustainability of the hospitality industry.

Moreover, previous studies have verified that the pandemic may impact PI [[12], [13], [14], [15], [16], [17], [18], [19]], along with social context, work environment, peer influence, professional regulations [20,21], and demographic factors [12,[22], [23], [24], [25]]. However, these questions have received little attention in TGPI and the tourism sector [26,27]. Jerez-Jerez et al. [28] pointed out that the focus on PI of hotel employees has been comparatively limited, even though increasing academic attention has been paid to the hospitality industry for its vital role in the tourism sector, not to mention the study on TGPI. Chen et al. [29] described that although TGs were powerful influencers of tourism, research on TGPI is relatively new and needs more academic attention. Given the vital role of PI in the tourism sector and the urgent need to clarify the current TGPI for the smooth resumption of tourism, research on TGPI from a practical perspective is necessary and imperative.

Few longitudinal studies on PI exist, particularly those observing changes around significant events like the COVID-19 pandemic. Existing studies often rely on retrospective data, potentially compromising authenticity due to changing contexts [12]. Therefore, a longitudinal study on TGPI is significant. This study proposed the research question, “What is the state of TGPI after the epidemic?” To clarify this, we must consider its current state, pre-pandemic changes, influencing factors, and impacts. The study proposed the following objectives:

Objective 1: Clarifying the current state of TGPI and its changes since 2019.

Objective 2: Determining the influence of demographic factors on TGPI pre- and post-pandemic.

Objective 3: Ascertaining the impact of TGPI and demographic factors on TGs’ professional decisions.

Given the limited literature in this field, this study contributes to understanding PI and TGPI in the hospitality and tourism sector by developing a definition and scale of TGPI based on TGs' practical work. It provides valuable research material to understand the pandemic's influence on PI, showing the change in TGPI and its influencing factors. In terms of practical contributions, this study may offer managerial implications to improve TGs' return-to-profession intention and inform policymaking for the tourism industry's post-pandemic development, as it provides information about the current state and influencing factors of TGPI and the influencing factor of TGs' post-returning intentions.

## 2 Literature review

This section presents academic findings on the definition of PI and TGPI, the influencing factors of TGPI and professional decision-making, and the insights of this study on these topics. Firstly, regarding the definition of PI, two perspectives exist: one views PI as a profession-specific identity, while the other perceives it as individuals' evaluation and recognition of their profession. This study adopted the latter perspective and developed the definition of TGPI, which reflects the extend TGs identify with their profession's values, emotions, and relationships, as well as their propensity to proactively enhance their professional competence.

Second, referring to the influence factors of PI, previous studies have indicated that the COVID-19 pandemic, demographic factors, social context, work environment, peer influence, professional regulations, job happiness [30], work-life balance [31], professional risk perception [11], and social support [11] may influence PI. These influencing factors of PI can be categorized into personal, family, institutional, and social factors, according to Mao et al. [25]. This study selected the pandemic, gender, education, certificate level, work age, income, and social insurance as potential influencing factors.

Third, the factors influencing professional decision-making are complex and can be categorized into three categories: readiness, orientation, and information, according to Kulcsár et al. [32]. In this study, several demographic factors and PI were chosen as the independent variables as potential influencing factors. The specific academic research achievements and elaborations are as follows.

### 2.1 The definition of PI

**Fig. 1:**
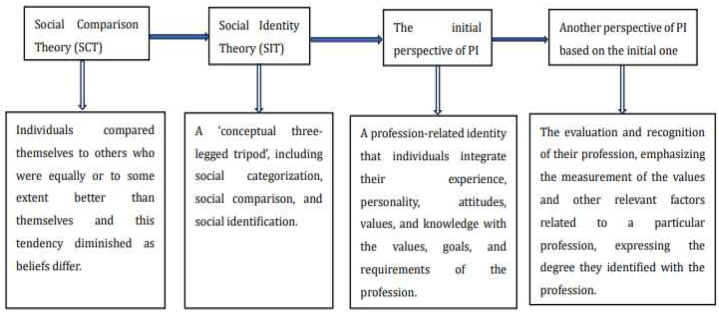
The development trajectory of PI.

There are two perspectives on the definition of PI: one views PI as a profession-specific identity, while the other perceives it as individuals' evaluation and recognition of their profession. The former perspective originated from the social identity theory (SIT) of Tajfel [33], which developed based on the comparison theory (SCT) of Festinger [34]. The latter perspective evolved from the former one. Fig. 1 illustrates the evolution of PI for clarity.

The first perspective was proposed based on SIT derived from SCT. The SCT of Festinger [34] argued that individuals compared themselves to others of equal or superior standing, with the tendency to equate with others decreasing as beliefs diverged [34]. Based on the SCT theory, Tajfel [33] developed the concept of “in-group” and “out-group(s),” referring to group members and non-members, respectively. Later, Tajfel [35] expanded his theory to describe the psychological and social process of intergroup relations in various contexts. He argued that social identity fundamentally concerns the societal recognition of individuals. Tajfel [36] further refined his theory, defining it as a “conceptual three-legged tripod” encompassing social categorization, social comparison, and social identification [36]. Based on SCT and SIT, the concept of PI was initially proposed in the teaching profession and subsequently extended to other professions [37,38]. Scholars adopting this perspective view PI as a profession-related identity, integrating individuals’ experience, personality, attitudes, values, and knowledge with the values, goals, and requirements of the profession (such as skills, ethics, and knowledge) [16,[37], [38], [39]].

The second perspective views PI as one's evaluation and recognition of their profession. This perspective emphasizes measuring values and other relevant factors related to a specific profession, expressing the degree of identification with the profession [5,[40], [41], [42], [43]]. For instance, Mancini et al. [42] viewed PI as one's awareness of their profession and identification with the professional groups they belong to. Yu et al. [5] defined PI as a progressive, dynamic process involving professional cognition, appraisal, and emotion. Similarly, Fitzgerald [43] viewed PI as an individual's evaluation of the goal, social value, and other factors associated with the profession.

Tajfel [33] emphasized the close relationship between the two perspectives, highlighting the significance of the consequences of group membership for individuals. If a group enhances one's social identity, membership is retained; otherwise, it may be relinquished. This implies that professional identity impacts cognition and self-esteem, prompting individuals to reassess their profession. On this basis, the second perspective of PI was formed.

### 2.2 The definition of TGPI

This study defined TGPI based on the perspective that PI is the evaluation and recognition of their profession. For instance, Wei and Huang [22] viewed TGPI as an interactive process of self-knowledge and positioning with others. Marhuenda et al. [44] argued that the sense of belonging to the group was vital in improving PI for tourism staff. Zhang [44] argued that TGPI was the degree to which TGs identified with tour guiding work. Pan et al. [45] defined TGPI as the extent to which TGs identified with their profession. Hence, this study summarized TGPI as the level of TGs' recognition of their profession.

Regarding the structure of TGPI, this study draws inspiration from the related literature about PI in the tourism sector, which is mainly concerned with the profession's values, emotions, relationships, and behavior. For instance, Peng & Li [46] insisted that the PI of Chinese hotel interns included professional values, professional belonging, professional behavioral inclination, professional will, and expectations. Van Dick et al. [47] argued that organizational identity, a unique PI, consisted of cognition, emotion, evaluation, and behavior. Marhuenda et al. [44] argued that the sense of belonging to the group was vital to improving the PI of tourism staff, emphasizing the importance of professional relationship identity. The limited literature about TGPI also pays close attention to professional-related values, emotions, relationships, and behavior. For instance, Zhang [44] regarded professional emotion and professional behavior as two dimensions of TGPI. Yu et al. [5] considered that TGPI was composed of professional cognition, professional appraisal, and professional emotion. Wei and Huang [22] emphasized the importance of professional relationships when they defined TGPI.

All these studies closely addressed four aspects: professional value, professional emotion, professional relationship, and professional behavior tendency. Accordingly, this study defined the TGPI as the degree to which TGs are identified with the tour guiding profession. Specifically, TGPI is the extent to which TGs identify with their profession's values, emotions, and relationships and the extent to which they are inclined to take the initiative to improve their professional competence, which consists of four dimensions of TGPVI, TGPEI, TGPRI, TGPBT.

(1) TGPVI is the extent to which TGs identify with the value of being a TG and the value of the TG profession.
(2) TGPEI is the degree to which TGs identify with the emotion attached to the tour guiding work, which arises from the practice of actual work and is a comprehensive emotion for the profession, containing what the TG has paid and gained in the tour guiding life.
(3) TGPRI is the degree to which TGs identify with the relationships with people involved in the TG profession, including relationships with other TGs, tourists, and tourism enterprises.
(4) TGPBT is the degree to which TGs tend to improve their ability to lead tours, such as broadening their knowledge base, learning new skills, and participating in various professional skills competitions and TG grade tests to serve tourists better.

### 2.3 The influence factors of PI

Some researchers have confirmed that social context, work environment, peer influence, and professional regulations impact PI [20,21]. Besides, other scholars found that job happiness [30], work-life balance [31], professional risk perception [11], and social support [11] may influence PI as well as demographic factors [12,[22], [23], [24], [25]].

This study chose gender, education, certificate level, work age, income, and whether they have social insurance as possible influencing factors and verified whether they impact TGPI, as several studies have shown that these factors impact TGPI. For instance, Wei and Huang [22] demonstrated that economic income and education impacted TGPI. Jin [24] found that gender significantly affects the PI of tourism students. Tang et al. [12] discovered that gender influences the PI of nursing students. Similarly, Mao et al. [25] found that personal factors, including age, gender, and years of study or years of work, influence the PI of nursing students.

### 2.4 Impact of the COVID-19 pandemic on PI

The COVID-19 pandemic has both positive and negative impacts on PI. However, there is a relative scarcity of literature on the effects of the pandemic on PI in the tourism sector, which is one of the reasons why this study focuses on the impact of the COVID-19 pandemic on PI.

Some studies indicated that the pandemic positively impacted PI; for instance, Stetson et al. [20] found that significant crises, such as the COVID-19 pandemic, shaped the identity of specific professions involved in the response. Other scholars have demonstrated that the PI level of medical students increased during the pandemic because of their engagement in pandemic-related health education and volunteering activities [12,18,19].

However, other scholars have shown that the COVID-19 pandemic had a significant adverse effect on PI. El-Soussi [48] found that as the teaching format shifted from face-to-face to online instruction due to the pandemic, the PI of teachers went through some precarious phases. Li et al. [11] discovered that the PI of medical professionals was reduced due to the professional risks they faced during the pandemic.

### 2.5 Effect of PI on professional decisions

As an essential variable to evaluate one's professional state, PI is closely associated with individuals' professional decisions. Hong [9] described PI as crucial in understanding individuals' professional states and decisions. The researchers found a close association between PI and job retention; individuals with higher levels of PI were more significantly committed to their profession [14,15]. Moreover, extensive literature suggests that PI negatively impacts turnover intention [13,16,[49], [50], [51]]. For instance, Wang et al. [13] confirmed that PI significantly negatively affected the turnover intention of China's hotel employees. Tsai and Chen [51] discovered that hospitality employees with a more robust PI were less likely to switch to other industries. Van der Heijden et al. [49] argued that PI significantly negatively influenced professional mobility intention.

Through the systematic review of the relevant literature, this study addresses several research gaps in the limited material on TGPI from different perspectives. First, this study advances knowledge of PI and TGPI in the hospitality and tourism industry by creating a definition and TGPI scale based on TGs' actual work from a fresh angle. Additionally, considering the scarcity of longitudinal studies in this field, this longitudinal study demonstrated the shift in TGPI and the change in its influencing elements under the pandemic, thereby providing invaluable research material to comprehend the impact of the pandemic on PI.

## 3 Methodology

### 3.1 Study design and settings

This study used a quantitative methodology to guarantee that the findings can be applied to the entire population, including comparative analysis (*t*-test and one-way ANOVA) and binary logistic regression [52]. Data were gathered online from China nationwide through the Questionnaire Star, an esteemed online survey software in China, due to the COVID-19 pandemic. To ensure the credibility of research results, participants were restricted to the TGs with authentic experience in tour guiding.

### 3.2 Sampling technique and data collection

A cross-sectional survey with convenience sampling was used for this study. Etikan et al. [53] argued that it is impractical to include every subject because the population is almost finite, which is the rationale for most researchers using convenience sampling. To conduct convenience sampling, we distributed the questionnaire to the WeChat groups of first-line TGs with authentic experience in tour guiding; the survey was conducted on four national WeChat groups with TGs from all over China. Each group comprised around 200–400 members. The author was a TG from 2012 to 2017 when she met many TGs and joined several TG WeChat groups, thereby adding credibility to the research results.

This longitudinal study contained two research studies: pre- and post-pandemic. The first research was conducted between September and October 2019, before the pandemic outbreak. A total of 422 questionnaires were collected, among which 370 were valid. The second research was conducted from May to June 2022, three years after the outbreak of the pandemic, to investigate the current state of TGPT in the context of China resuming inter-provincial travel in May 2022 after a shutdown of nearly three years. In the second research, 398 questionnaires were collected, with 342 valid questionnaires left. Invalid forms are those in which respondents provide consistent answers to all questions or fill in within an unreasonably short time.

To minimize experimental errors, this study conducted two surveys using the same research channels and methods through Questionnaire Star; the same questionnaire was distributed to the same WeChat group in 2019.

This data collection method is widely used in longitudinal studies. For instance, to clarify the changes in levels of psychological impact, stress, anxiety, and depression during the pandemic, Wang et al. (2020) conducted two surveys from January 31 to February 2, 2020 (first survey, n = 1210) and February 28 to March 1, 2020 (second survey, n = 861), with the same questionnaire, and then made a comparison between the data. Moreover, to research the changes in the mental health of adults in France due to the general population lockdown implemented in the face of the COVID-19 epidemic, Ramiz et al. (2021) conducted research in 2020 and compared the data with the average results from 2014 to 2019 using the same questionnaire.

### 3.3 Questionnaire tools and instruments

The questionnaire consisted of demographic factors and the TGPI scale. The demographic factors included gender, work age, certificate level, income, education level, and social insurance. In the survey processed in 2022, many TGs indicated they would not return to the position after the pandemic, while some responded that they would return. Thus, this study added Question 3 to investigate whether they will return to their post and its influence factors. According to the TGPI scale, this study used a Likert-type scale, ranging from one (strongly disidentify) to five (strongly identify), which consists of 14 questions across four dimensions (TGPVI, TGPEI, TGPRI, and TGPBT). We used the same questionnaire in both phases but added the question “Will you return to the tour guide profession after the pandemic?” with the answer of yes or no in the second phase for the research need.

The task of developing the TGPI scale went through four steps. In the first step, four dimensions were generated after scrutinizing the relevant literature mentioned above (Table 1). Then, a pool of items associated with these dimensions was designed in the second step. After that, the developed TGPI scale was critically reviewed by experienced TGs and authoritative professors specializing in tourism in the third step. Finally, the incorrect items were removed, and the necessary modifications were made to ensure that the scale aligned with the actual work practices and characteristics of TGs. The final TGPI scale and the source literature are indicated in Table 1.

**Table 1:** Measurement items of the TGPI scale.

Dimensions	Items	Sources Literature
TGPVI	I find the profession of TG very rewarding.	Yu et al. [5] Johnson et al. [31] Peng and Li [37]
	I feel that the profession of TGs has improved my ability, broadened my horizon, and realized my self-worth.	
	Although there are now many virtual TG, I think virtual TG still can't replace the value of physical TG.	
TGPEI	I am proud to be a TG.	Zhang [48] Yu et al. [5] Johnson et al. [31]
	I love the profession of TG.	
	I have a deep affection for the profession of TG	
	I am very committed to the profession of TG.	
TGPRI	The other TGs are very friendly to me; we are very united and get along well.	Wei and Huang [34] Marhuenda et al. [35] Peng and Li [37]
	The travel agency owners are very nice to me, and we cooperate well.	
	I get along well with tourists.	
TGPBT	I will proactively seek feedback from tourists to improve my services.	Zhang [48] Hao (2011) Van Dick et al. [38] Peng and Li [37]
	I will spend my spare time to learn about knowledge about how to lead tourist groups to improve my professional capabilities.	
	I will participate in various TG competitions and TG rating exams to improve my professional capabilities.	
	I will learn the experience and knowledge from outstanding TGs.	

### 3.4 Data analysis

To achieve the research objective “clarifying the current state of TGPI and its changes compared to the pre-pandemic in 2019,” this study was conducted on the 2019 and 2022 samples. To achieve the research objective “determining the influence of demographic factors on TGPI before and after the pandemic,” the T-test and one-way ANOVA method was used. To achieve the research objective “ascertaining the influence of TGPI and the demographic factors on professional decisions of TGs,” a binary logistic regression was conducted as the dependent variable. Liang et al. [54] argued that logistic regression, which yields odd rates for each variable, was suitable for discrete outcomes under a set of binary predictor variables. It has been used in many studies in the tourism field [55]. This study used the decision of TGs whether they would return to their post as the dependent variable with choices of yes or no while selecting the demographic factor and the four TGPI dimensions as the independent variables. The mathematical calculation principle of the binary logistic regression model is shown as follows [56]:

The probability of deciding to return to the position can be simulated using a logistic regression model, as shown in Equation (1).

(1) $$\pi_{i}=\frac{{\mathrm{e}}^{z_{i}}}{1+{\mathrm{e}}^{z_{i}}}$$

Here, πi represents the probability of the TG deciding to return to the position, Zi represents the utility of choice to return to the position, and Eq. (2) is as follows.

(2) Zi = B0 + B1X1 + B2X2 + ⋯ + BNXN

Xi represents the independent predictor variables, and Bi represents the model coefficients of the respective independent predictor variables, which can be estimated using the maximum likelihood method. Binary logistic models predict the probability of variations (increase or decrease) in these variables for every unit variation in the predictor variables. The exponential function in Eq. (3) expresses the estimation of the odds ratio (OR) obtained by dividing the probability of the occurrence of the outcome event by the probability of its non-occurrence.

(3) $$OR={\mathrm{e}}^{B_{i}}$$

in this study, most explanatory variables, being categorical, were represented as dummy variables. We used n-1 dummy variables to represent an explanatory variable with n categories. We selected a baseline category for each variable and then compared it against all remaining categories to determine the odds ratios. To ensure the validity of the results, we checked for multicollinearity before building the regression model; checking the correlation between variables is a simple and effective way. If the correlation between independent variables is too high, they may have a high probability of covariance. This study used the VIF test to identify multicollinearity [57,58], with 3.3 as the VIF threshold value [59].

### 3.5 Ethical clearance

This study was approved by the School of Culture and Tourism, Henan University, and the ethical approval number is 2024-010LLWL-003. Before data collection, participants were informed by the authors about the goal of the study, the procedure for gathering data, and the intended use of the data. It was made clear that the survey was anonymous, and no identifiable information was obtained. All participants completed the questionnaire voluntarily and could opt out of data collection anytime. Additionally, they were advised that answering the questionnaire would be deemed a written agreement.

## 4 Results

### 4.1 Factor analysis results for TGPI scales

This study conducted an EFA to identify TGPI dimensions using the principal component method with orthogonal varimax rotation. It eliminated items under more than one dimension with close factor loadings and items with factor loadings below 0.5. The EFA was run first on the 2019 sample and then on the 2022 sample. After that, the 2019 and 2022 samples obtained the final four factors with the cumulative explained variances of the total variances of 69.785 % and 70.124 %, respectively. Moreover, the KMO of both years is 0.911 and 0.892, with the Bartels test of sphericity being 2721.702 and 2228.152, respectively. Both samples yielded a significant result of 0.000 with 91 degrees of freedom.

The detailed results of EFA for 2019 and 2022 are presented in Table 2 and Table 3, demonstrating the measures of composite reliability of the rotated factor loadings, Cronbach's alpha of each factor, and cumulative explained variances. As the TGPI scale is self-developed based on literature and the actual practice of TGs, this study chooses 0.6 as the criteria to determine the internal reliability of each factor, as Abdullah et al. [60] argued that the research tool is reliable if Cron's alpha value is > 0.6; however, it is not reliable if the value is < 0.6, which was supported by many other researchers [[61], [62], [63]].

**Table 2:** Factor analysis of TGPI of 2019 sample (N = 370).

Factor/Item	Ma	SD	Cronbach's a	Loading	% of variance
Factor 1: TGPBT			0.779		18.625
I will proactively seek feedback from tourists to improve my services.	3.84	0.736		0.696	
I will spend my spare time to learn about knowledge about how to lead tourist groups to improve my professional capabilities.	4.08	0.674		0.822	
I will participate in various TG competitions and TG rating exams to improve my professional capabilities.	3.72	0.851		0.738	
I will learn the experience and knowledge from outstanding TGs.	4.27	0.668		0.655	
Factor 2: TGPEI			0.789		37.133
I am proud to be a TG.	3.98	0.726		0.642	
I love the profession of TG.	3.95	0.675		0.695	
I have a deep affection for the profession of TG	3.67	0.902		0.745	
I am very committed to the profession of TG.	3.82	0.769		0.840	
Factor 3: TGPRI			0.717		54.498
The other TGs are very friendly to me; we are very united and get along well.	3.65	0.853		0.829	
The travel agency owners are very nice to me and we cooperate well.	3.93	0.805		0.796	
I get along well with tourists.	3.43	1.004		0.799	
Factor 4: TGPVI			0.651		70.124
I find the profession of TG very rewarding.	4.13	0.806		0.753	
I feel that the profession of TGs has improved my ability, broadened my horizon, and realized my self-worth.	4.18	0.765		0.779	
Although there are now many virtual TG, I think virtual TG still can't replace the value of physical TG.	4.31	0.771		0.770	

KMO: 0.911; Bartels: 2721.702; Total % of variance: 69.785; DF:91; Sig:0.000.

Note: *p < 0.05; **p < 0.01; ***p < 0.001; ns = not significant.

**Table 3:** Factor analysis of TGPI of 2022 sample (N = 342).

Factor/Item	Ma	SD	Cronbach's a	Loading	% of variance
Factor 1: TGPBT			0.779		18.625
I will proactively seek feedback from tourists to improve my services.	3.84	0.736		0.696	
I will spend my spare time to learn about knowledge about how to lead tourist groups to improve my professional capabilities.	4.08	0.674		0.822	
I will participate in various TG competitions and TG rating exams to improve my professional capabilities.	3.72	0.851		0.738	
I will learn the experience and knowledge from outstanding TGs.	4.27	0.668		0.655	
Factor 2: TGPEI			0.789		37.133
I am proud to be a TG.	3.98	0.726		0.642	
I love the profession of TG.	3.95	0.675		0.695	
I have a deep affection for the profession of TG	3.67	0.902		0.745	
I am very committed to the profession of TG.	3.82	0.769		0.840	
Factor 3: TGPRI			0.717		54.498
The other TGs are very friendly to me; we are very united and get along well.	3.65	0.853		0.829	
The travel agency owners are very nice to me and we cooperate well.	3.93	0.805		0.796	
I get along well with tourists.	3.43	1.004		0.799	
Factor 4: TGPVI			0.651		70.124
I find the profession of TG very rewarding.	4.13	0.806		0.753	
I feel that the profession of TGs has improved my ability, broadened my horizon, and realized my self-worth.	4.18	0.765		0.779	
Although there are now many virtual TG, I think virtual TG still can't replace the value of physical TG.	4.31	0.771		0.770	

KMO: 0.892; Bartels: 2228.152; Total % of variance: 70.124; DF:91; Sig:0.000.

Note: *p < 0.050; **p < 0.010; ***p < 0.001; ns = not significant.

### 4.2 The score of TGPI and its dimensions in 2019 and 2022

Table 4 presents the scores of the total and four dimensions of TGPI in two stages. It shows that the scores of TGPEI (*p* < 0.01), TGPRI (*p* < 0.001), and TGPBT (*p* < 0.01) reduced significantly in 2022 compared to the pre-pandemic in 2019, while there was no significant change in TGPVI.

**Table 4:** The score of TGPI and its dimensions in 2019 and 2022.

	2019(N = 370) Mean ± SD	2022(N = 342) Mean ± SD	t- Value	P Value
TGPVI	4.260 ± 0.634	4.206 ± 0.648	1.138	0.255
TGPRI	4.069 ± 0.757	3.668 ± 0.777	6.968***	**0.000**
TGPEI	4.098 ± 0.623	3.857 ± 0.626	5.153**	**0.000**
TGPBT	4.180 ± 0.524	3.977 ± 0.581	4.887**	**0.000**
TGPI	4.152 ± 0.524	3.927 ± 0.513	5.777***	**0.000**

### 4.3 Demographic characteristics and the scores of TGPI on demographic factors

Table 5 displays the demographic characteristics of TG participants. The majority of the respondent TGs were females (77.8 % in 2019, 64.6 % in 2022), without social insurance (59.5 % in 2019, 52.6 % in 2022), possessing a college-level education (55.7 % in 2019, 44.2 % in 2022), and had over ten years tour guiding experience (40 % in 2019, 53.8 % in 2022). In terms of income, most of the samples of 2019 earned 30,000–60,000 RMB per year (33.8 %), while in 2022, most earned under 30,000 RMB (33.8 %). Regarding TGs’ professional choice, 267 tour guides said they would return to their posts, while 75 opted not to.

**Table 5:** Demographic characteristics and the scores of TGPI on demographic factors.

Demographics	2019(n = 370)				2022(n = 342)				
	n (%)	Mean ± SD	t/F	p	n (%)	Mean ± SD	t/F	p	post-hoc
**Gender**			−1.135	0.257			−0.776	0.439	
Male	82(22.2)	4.094 ± 0.555			121(35.4)	3.985 ± 0.621			
Female	288(77.8)	4.168 ± 0.514			221(64.6)	3.944 ± 0.444			
**Insurance**			2.495	0.013			0.114	0.910	
Yes	150(40.5)	4.233 ± 0.485			162(47.4)	3.930 ± 0.538			
No	220(59.5)	4.096 ± 0.543			180(52.6)	3.924 ± 0.491			
**Income/a year**			0.386	0.763			3.462	0.017	
Under 30,000 RMB	109(29.5)	4.118 ± 0.495			102(29.8)	3.892 ± 0.491			0.002**
30,000–60,000 RMB	125(33.8)	4.175 ± 0.515			65(19)	3.8907 ± 0.613			0.040
60,000–100,000 RMB	90(24.3)	4.136 ± 0.591			85(24.9)	3.93 ± 0.500			
Over 100,000 RMB	46(12.4)	4.200 ± 0.482			90(26.3)	4.061 ± 0.445			0.089
**Work age**			1.315	0.269			2.154	0.093	
Below 2 years	51(13.8)	4.040 ± 0.496			35(10.2)	3.772 ± 0.479			
3–5 years	64(17.3)	4.114 ± 0.500			48(14)	3.861 ± 0.515			
5–10 years	107(28.9)	4.161 ± 0.512			75(21.9)	3.902 ± 0.598			
Over 10 years	148(40)	4.200 ± 0.549			184(53.8)	3.984 ± 0.476			
**Grade**			2.788	0.063			1.407	0.246	
Primary	251(67.8)	4.12 ± 0.531			185(54.1)	3.921 ± 0.509			
Middle Level	70(18.9)	4.163 ± 0.480			62(18.1)	3.850 ± 0.586			
Advanced	49(13.2)	4.301 ± 0.526			95(27.8)	3.989 ± 0.467			
**Education**			1.316	0.269			0.520	0.669	
High School	32(8.6)	3.951 ± 0.610			18(5.3)	3.939 ± 0.560			
College	206(55.7)	4.272 ± 0.510			151(44.2)	3.962 ± 0.510			
Bachelor	118(31.9)	4.139 ± 0.510			133(38.9)	3.905 ± 0.498			
Master and above	14(3.8)	4.165 ± 0.538			40(11.7)	3.864 ± 0.561			
**Whether return to position**									
Yes					267(78.1)				
no					75(21.9)				

Note: *p < 0.050; **p < 0.010; ***p < 0.001.

Besides, Table 5 presents a gender disparity among tour guides, reflecting the situation in China, where there are far more female tour guides considerably outnumber their male counterparts. This skewed gender distribution is corroborated by several studies. For instance, the study by Qi &Ping-fang [64] reported 57 male TGs and 127 female TGs. Hu, W.W [65]. found 82 male and 285 female TGs. Zhang, A.P [66]. supported this trend, with 60 male and 195 female TGs.

**Fig. 2:**
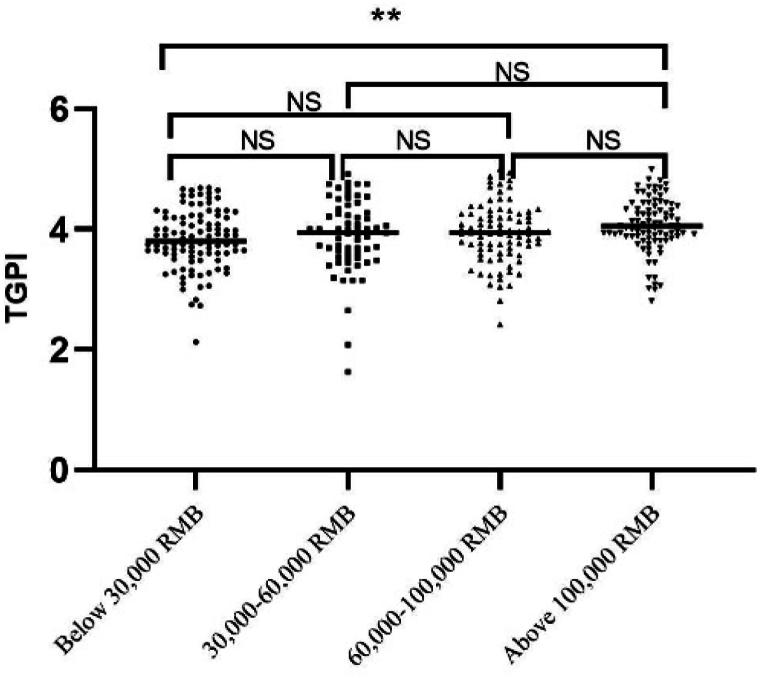
The scatterplot of the analysis of variance of TGPI among income. Note: *p < 0.050; **p < 0.010; ***p < 0.001; ns = not significant.

In addition, Table 5 also reveals a shift in the impact of demographic factors on TGPI between 2019 and 2022. Before the COVID-19 pandemic in 2019, only social insurance significantly affected TGPI. TGs with social insurance had significantly higher TGPI levels without significant differences in gender, income, work age, certificate level, and education. Three years after the outbreak of the pandemic in 2022, payment has become the only factor influencing TGPI significantly. To visualize disparities among income groups, a scatterplot of the analysis of variance (ANOVA) was generated using GraphPad Prism 9.0 (Fig. 2). The plot distinctly demonstrates variations in TGPI levels between individuals with annual incomes below 30,000 RMB and those with earnings exceeding 100,000 RMB.

### 4.4 Factors influencing decisions of TGs

Applying binary logistic regression requires no significant multicollinearity; a VIF value below 3.3 was considered acceptable [67]. Table 6 indicates that all VIF values in the model were less than 3.3, suggesting no significant multicollinearity and ensuring the reliability of the binary logistic regression model.

**Table 6:** The VIF of variables.

Variables	TGPVI	TGPRI	TGPEI	TGPBT	gender	education	work-age	insurance	grade	income
VIF	1.462	1.554	1.940	1.838	1.075	1.262	1.351	1.078	1.361	1.220

**Fig. 3:**
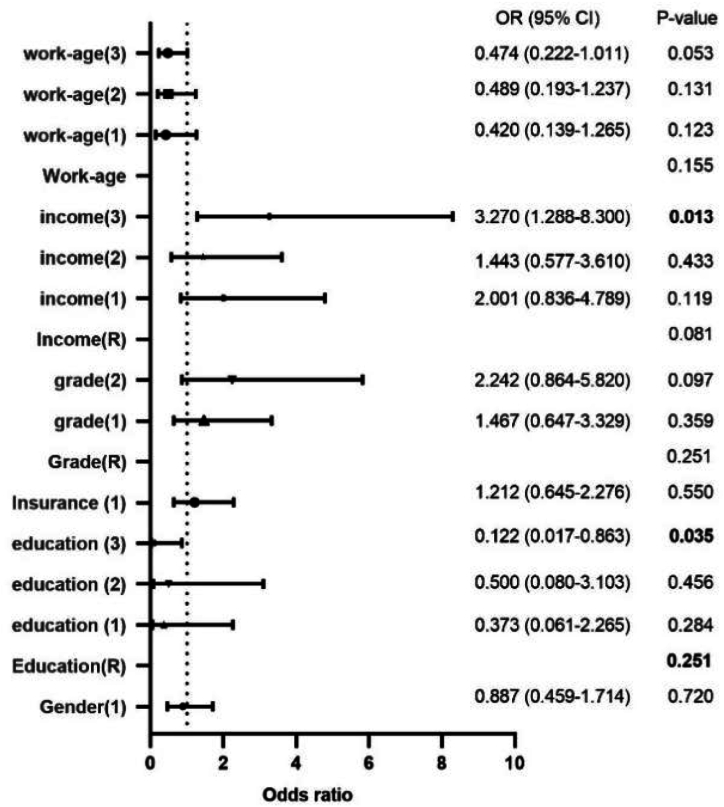
The result of binary logistic regression.

Table 7 presents the metrics of the binary logistic regression, showing a good model fit. The Omnibus tests for model coefficients are significant (chi-square value = 82.2, DF = 17, p = 0.000), which indicates that the overall model is statistically significant. Besides, the Hosmer and Lemeshow test indicates chi-square value = 7.612 (non-significant), DF = 8, and p = 0.472. It suggests that the model fits the data well, and there is no evidence of a lack of fit, supporting the adequacy of the model. In addition, the 2Log likelihood value is 277.597, while the values of Cox and Snell R Square and Nagelkerke R Square are 0.214 and 0.328, respectively, which are all by the regulations. Thus, all data indicate a good model fit with a high degree of data interpretation. This study presents the results of the binary logistic regression in Table 7. To provide an intuitive representation, we created a forest plot using GraphPad Prism 9.0 (Fig. 3).

**Table 7:** Result of the binary logistic regression.

	B	S.E,	Wald	Sig.	Exp (B)	95 % C.I. for Odds	
						Lower	Upper
Gender (1)	−0.120	0.336	0.128	0.720	0.887	0.459	1.714
Education(R)			10.062	0.018*			
education (1)	−0.987	0.921	1.149	0.284	0.373	0.061	2.265
education (2)	−0.694	0.932	0.555	0.456	0.500	0.080	3.103
education (3)	−2.104	0.998	4.443	0.035*	0.122	0.017	0.863
Work-age(R)			5.238	0.155			
work-age (1)	−0.868	0.563	2.378	0.123	0.420	0.139	1.265
work-age (2)	−0.716	0.473	2.284	0.131	0.489	0.193	1.237
work-age (3)	−0.747	0.387	3.735	0.053	0.474	0.222	1.011
Insurance (1)	0.192	0.322	0.357	0.55	1.212	0.645	2.276
Grade(R)			2.768	0.251			
grade (1)	0.383	0.418	0.842	0.359	1.467	0.647	3.329
grade (2)	0.807	0.487	2.751	0.097	2.242	0.864	5.820
Income(R)			6.72	0.081			
income (1)	0.694	0.445	2.427	0.119	2.001	0.836	4.789
income (2)	0.367	0.468	0.614	0.433	1.443	0.577	3.610
income (3)	1.185	0.475	6.215	0.013*	3.270	1.288	8.300
PVI	1.193	0.297	16.09	0.000***	3.296	1.840	5.904
PRI	0.570	0.242	5.547	0.019*	1.769	1.100	2.843
PEI	0.297	0.337	0.776	0.378	1.345	0.695	2.604
PBT	0.035	0.358	0.010	0.922	1.036	0.514	2.088
Constant	−6.363	1.779	12.788	0.000	0.002		

Omnibus tests for model coefficients: chi-square value = 82.2, DF = 17 and p = 0.000.

Hosmer Lemeshow's goodness of fit: chi-square value = 7.612, DF = 8 and p = 0.472.

Value of 2Log likelihood: 277.597; Cox and Snell R Square:0.214; Nagelkerke R Square 0.328.

Note: Gender (1) = female; Education(R) = high school, education (1) = college, education (2) = undergraduate, education (3) = postgraduate or above, Work-age(R)=< 2 years; work-age (1) = 3–5 years, work-age (2) = 6–10 years, work-age (3)= >10years, Insurance (1); Grade(R) = primary, grade (1) = middle level, grade (2) = advanced level; Income(R)=< 30,000 RMB, income (1) = 30,000–60,000 RMB, income (2) = 60,000–100,000 RMB, income (3) = Over 100,000 RMB.

The results of binary logistic regression indicate that gender, work age, certificate level, insurance, TGPEI, and TGPBT did not significantly influence TGs' decisions (Table 7 and Fig. 3). In contrast, TGPI (TGPVI, TGPRI), income, and education level significantly affected their choice. Specifically, for every unit higher in TGPVI, the probability of TGs returning to their post would increase by 3.296 times (*p* = 0.00); for every unit higher in TGPRI, the possibility of TGs returning to their position will increase by 1.769 times (*p* = 0.019); the possibility of TGs returning to the position with a master's degree or higher is only 0.122 times of TGs with a high school degree (*p* = 0.035); the probability of TGs who earn over 100,000 RMB annually returning to the post is 3.27 times of TGs whose income is below 100,000 RMB annually (*p* = 0.013).

## 5 Discussion

This quantitative study was conducted to provide an overview of the state of TGPI after the Covid-19 pandemic. To achieve this goal, we set three specific research objectives and employed the comparative analysis and binary logistic regression method. Our findings will enlighten researchers interested in PI study, particularly within the tourism sector, and those investigating the impact of the pandemic on work patterns by the Covid-19 pandemic. The results of the three research objectives were as follows:

(1) The current TGPI is at a medium level, reducing significantly compared to the pre-pandemic period in 2019. Besides, the three dimensions of TGPEI, TGPRI, and TGPBT of TGPI reduced significantly in 2022 compared to 2019, without significant change in the dimension of TGPVI.
(2) In the 2019 sample, social insurance was the only factor impacting TGPI; TGs with social insurance had significantly higher TGPI levels. No significant differences were detected in gender, income, work age, certificate level, or education. For the 2022 sample, however, income was the only factor significantly influencing TGPI; the TGPI levels of TGs with earnings over 100,000 RMB a year were considerably higher than those whose income is below 30,000 RMB per year.
(3) Gender, work age, certificate level, insurance, and TGPEI and TGPBT did not significantly influence the returning intentions of TGs. In contrast, TGPVI, TGPRI, income, and education significantly affected the choices of TGs.

First, according to the first result, TGPI is currently at a medium level, with a significant reduction compared to the pre-pandemic period in 2019. It aligns with El-Soussi's [48] findings that the COVID-19 pandemic has reduced the PI of teachers. However, research by Nie et al. [68] and Tang et al. [12] showed that the pandemic has enhanced the PI of nurses and medical students. This inconsistency could be attributed to the direct involvement of those professions in pandemic-related work, which has reinforced both their professional and individual value, resulting in improved PI. However, TGs have lost their jobs due to the pandemic and have been separated from their professions for a long time, negatively impacting their TGPI. Specifically, the interruption of connections between TGs, tourism companies, and tourists has decreased TGPRI. The long-term separation from the industry has led to a significant decrease in attachment to the profession, thus reducing the TGPEI significantly. Long-stagnant tourism has destroyed the confidence of TGs and led to a pessimistic view of their profession, leading to a low TGPBT. Due to its close relationship with TGs' self-worth identity, TGPVI remained unchanged, which indicates that TGs maintained a positive attitude toward the value of their profession.

Second, the second result showed that the only factor influencing TGPI in 2019 was social insurance, while in 2022, it was income. It is consistent with the findings of Wei and Huang [22] that the payment of TGs impacted the level of TGPI. Results of both years show that the factors that affected TGPI were related to finance: social insurance indirectly influenced it pre-pandemic in 2019, while it reflects on income directly affected it post-pandemic in 2022. It could be attributed to TGs bearing the entire burden of social insurance, as many tourism companies have gone bankrupt due to the pandemic, or since social insurance has only played a small role in changing the economic situation of TGs.

Third, as shown in the third result, TGPEI, TGPRI, income, and education level significantly influence the decision of TGs to return to their post after the pandemic. It aligns with the study of Lu et al. [15], which showed that PI has a salient effect on job retention; the higher the PI levels, the higher the commitment to a profession. Besides, it aligns with the findings of Hu et al. [14] and TÜRKSOY [16] that PI significantly negatively impacted turnover intention. According to the resource protection theory, the risk of losing a job during the pandemic might also impact TGPI, prompting them to seek alternative professions to avoid further resource loss in the future [11]. Besides, the result that TGs with a master's degree or above were more likely to leave the tour guiding profession aligns with the research of Brien et al. [17] that the hotel staff with higher education were more inclined to leave. There is a particular reason for this phenomenon in China's social context. With the spread of higher education in China, many professions, such as teachers, civil servants, state-owned enterprises, and foreign enterprises, require a higher level of education compared to the lower threshold for staff in the tourism sector, like attendants or TGs. Many TGs lost their jobs due to the pandemic, leading TGs with higher education to look for professions less affected by the pandemic. However, it is difficult for TGs with lower education levels to find an ideal job due to the less optimistic national economy. Therefore, they had to work for temporary employment and look forward to returning to the TG profession when the pandemic ends.

## 6 Implications

### 6.1 Theoretical implications

Theoretically, through literature review and real-world TG practices, this empirical study offers a definition and a scale of TGPI, thereby expanding the scope of PI research. Second, since the research on PI within the tourism sector is quite limited, this study provides valuable material for scholars interested in this topic. Third, this study also found changes in the influencing factors of TGPI pre- and post-epidemic, which will highlight the impact of the epidemic on employees' work status and understand the changes in employees' status after the epidemic. Fourth, this study showed that the TGPI, as well as some demographic factors, would have an impact on TGs' willingness to return to their positions after the epidemic, which will also have some insights for scholars who are interested in employees’ professional decisions and turnover intention after the pandemic.

### 6.2 Practical implications

This study will shed some light on understanding the working status of TGs and their professional decisions, which will be helpful for tourism business operators to manage their employees and cultivate their talents. Besides, it has practical inspiration for tourism government departments to formulate relevant tourism development policies after the epidemic and the construction of tourism talent. To increase the probability of TGs returning to their posts, targeted improvement suggestions are as follows.

First, it is imperative to carry out various activities to promote exchanges between industries to improve the TGPRI and the TGPEI. For example, multiple events should be held to promote the exchange of friendship between guides, encourage travel agencies to conduct tourism line pre-sale activities, and impel tourist attractions to organize free tour guide stepping activities.

Second, it is essential to give social support to TGs due to its significant positive role in improving the TGPI and the possibility of returning to their positions. Social support refers to the degree to which a person feels valued, cared for, and respected by one's family or others. It consists of objective support (financial support, free online training, subsidies, labor rights, and protection of interests) and emotional support (condolences, care, and psychological counseling) [69]. For example, El-Soussi [48] argued that policymakers should provide both online teaching and training and social support for teachers to promote the positive development of their PI during the pandemic. Li et al. [11] also elaborated that social support mitigated the effects of professional risk on career mobility intentions and PI of medical staff.

Third, the government, tourism companies, and the media should give TGs the appropriate social support-not only materially, such as financial support, free online training, subsidies, labor rights, and protection of interests, but also emotionally, such as condolences, care, and psychological counseling.

## 7 Limitations

This study, like any other, has certain limitations. First, the sample was solely drawn from WeChat groups of TGs, and the limited sample size may affect the results. Future research can increase the number of respondents and explore multiple channels. Second, regarding the binary logistic regression model, this study only chose the demographic variables and the four dimensions of TGPI as independent variables, aiming to explore their relationship with the dependent variable. In future studies aiming to accurately predict a specific tour guide's professional mobility intention, more independent variables, such as professional risk perception and social support, should be considered and added, as Li et al. [11] showed that those variables could impact the results. Third, variables such as motivation [70] and engagement of employees [13] may affect the relationship between tour guides' professional identity and professional decisions, potentially impacting the results.

## 8 Conclusion

Based on previous research and the actual TG practices, this study developed the definition and the scale for TGPI. Via a quantitative approach, this study clarifies the current status of the TGPI after the epidemic, including the current score changes and shifts in influencing factors compared to those in the pre-epidemic period. Additionally, TGs’ return intention to their positions was also explored, as well as the impact of TGPI and demographic factors on this decision. The contribution of the current study is twofold. Theoretically, it contributes to the study of PI within the field of tourism and the influence of the pandemic on the work status and career decisions of employees. Practically, it provides insights for tourism enterprises striving to retain talent post-epidemic and offers guidance for government departments involved in post-epidemic tourism policymaking and talent acquisition strategies. Enterprises and government departments are urged to attentively address this concern, develop comprehensive policies for retaining tourism talent, devise pragmatic and efficacious initiatives, and establish proficient tourism talent teams, all aimed at fostering the sustainable advancement of the tourism sector.

## Data availability statement

Data will be made available on request.

## Funding statement

This work was supported by China National Social Science Foundation [grant number 19BGJ007]; Program for Humanities and Social Science Foundation of the Ministry of Education in China [grant number 22YJC760118]; Major scientific research projects in Colleges and Universities in Guangdong [grant number 2021ZDJS122]; Philosophy and Social Science Project of Guangdong Province [grant number GD24CGL26].

## Additional information

No additional information is available for this research article.

## CRediT authorship contribution statement

**Wenwen Hu:** Writing – original draft, Validation, Software, Investigation, Formal analysis, Data curation, Conceptualization. **Qing Yuan:** Writing – review & editing, Validation, Project administration, Methodology, Funding acquisition. **Yaxi Wang:** Visualization, Software, Formal analysis. **Nan Chen:** Writing – review & editing, Writing – original draft, Validation, Supervision, Investigation, Funding acquisition, Conceptualization.

## Declaration of competing interest

The authors declare the following financial interests/personal relationships which may be considered as potential competing interests:Yuan Qing reports financial support was provided by Ministry of Education in China. Chen Nan reports financial support was provided by China National Social Science Foundation. WenWen Hu reports financial support was provided by Philosophy and Social Science Project of Guangdong Province. If there are other authors, they declare that they have no known competing financial interests or personal relationships that could have appeared to influence the work reported in this paper.
